# Thyroid volume’s influence on energy deposition from ^131^I calculated by Monte Carlo (MC) simulation

**DOI:** 10.2478/v10019-011-0008-5

**Published:** 2011-03-29

**Authors:** Ali Asghar Mowlavi, Maria Rosa Fornasier, Mario de Denaro

**Affiliations:** 1 Department of Medical Physics, A.O.U. “Ospedali Riuniti di Trieste”, Trieste, Italy; 2 Physics Department, School of Sciences, Sabzevar Tarbat Moallem University, Sabzevar, Iran

**Keywords:** thyroid gland, ^131^I radionuclide, total energy deposition, MCNPX code

## Abstract

**Background:**

It is well known that the success of the radiomethabolic ^131^I treatment of hyperthyroidism could depend on the absorbed dose to the thyroid. It is, thus, very important to calculate the individual radiation dose as accurately as possible for different masses of thyroid lobes. The aim of this work is to evaluate the influence of thyroid volume on the energy deposition from beta and gamma rays of ^131^I by Monte Carlo (MC) simulation.

**Materials and methods.:**

We have considered thyroid lobes having an ellipsoidal shape, with a density of 1.05 g/ cm^3^ and the material composition suggested by International Commission on Radiological Protection (ICRP). We have calculated the energy deposition of ^131^I rays for different volumes of thyroid lobes by using the MCNPX code, with a full transport of beta and gamma rays.

**Results and conclusions.:**

The results show that the total energy deposition has a significant difference, till 11%, when the lobe’s volume varies from 1 ml to 25 ml, respect to the value presented in MIRDOSE for a 10 g sphere. The absorbed energy fraction increases by volume, because the increasing volume to surface ratio of ellipsoidal lobe causes the decrease of beta ray fraction escaping from the lobe.

## Introduction

Thyroid gland consists of two linked lobes and is located in the middle of the low neck, overlying the trachea. Radioactive iodine ^131^I has become the most widely used therapy for patients with hyperthyroidism due to Graves’ disease.^1^ This kind of therapy has largely replaced surgery as the definitive treatment for such benign disease in contrast with malignant ones^2^–^4^, because it is easier than surgery to perform and has proved to be more effective. A number of dosing regimens have been proposed, ranging from those based on thyroid volume evaluation and Iodine test-activity uptake determination – for high precision dosimetry –, to large, fixed activities of ^131^I administration, intended to cause hypothyroidism soon after treatment.^1^–^3^ Physicians generally determine the ^131^I activity on an empirical basis: the decision is based on the volume of the thyroid evaluated by scintigraphy, SPECT, MRI or ultrasonic methods and, sometimes, on the basis of ^131^I/^123^I test-activity uptake at 24 hours post-administration.^5^

It is well known that the success of this therapy could depend on the absorbed dose to the thyroid: it is thus very important to calculate the individual radiation dose as accurately as possible for different mass of thyroid lobe. Many authors have developed algorithms for the calculation of the radiation absorbed dose to a target organ, starting from a basic absorbed dose rate equation represented by the Medical Internal Radiation Dose (MIRD) models.^6^ Traino *et al.* evaluated the influence of the volume reduction on the calculation of the absorbed dose to the thyroid by presenting a mathematical model.^1^ The aim of this work is to evaluate the influence of thyroid volume on the energy deposition from ^131^I by Monte Carlo (MC) simulation.

## Materials and methods

MCNPX is a general purpose, continuous and discrete energy, generalized-geometry, time-dependent code to simulate particles transport, based on Monte Carlo method. It is an extremely useful tool for radiations transport simulation and tracks about 40 particles including some light ions.^7^ The code is written in Fortran 90 and contains flexible source and tally options; it utilizes the latest nuclear cross section libraries with a data library of photons cross-section ranging from 1 keV to 100 GeV.

This code has been used to calculate the energy deposition from beta and gamma rays of ^131^I for a thyroid lobe of ellipsoidal shape, with the major axis two times of the minor axis, 1.05 g/cm^3^ density and with a mass varying from 1 g to 25 g.

In running MCNPX code, we have considered the “full transport” for both gamma and beta rays; that is, we have considered that beta rays do not deposit their energy in a starting point, but they undergo many Coulomb interactions, so that a significant portion of their energy, near the surface of lobes, escapes and is stored out of the thyroid lobes.

**FIGURE 1.**
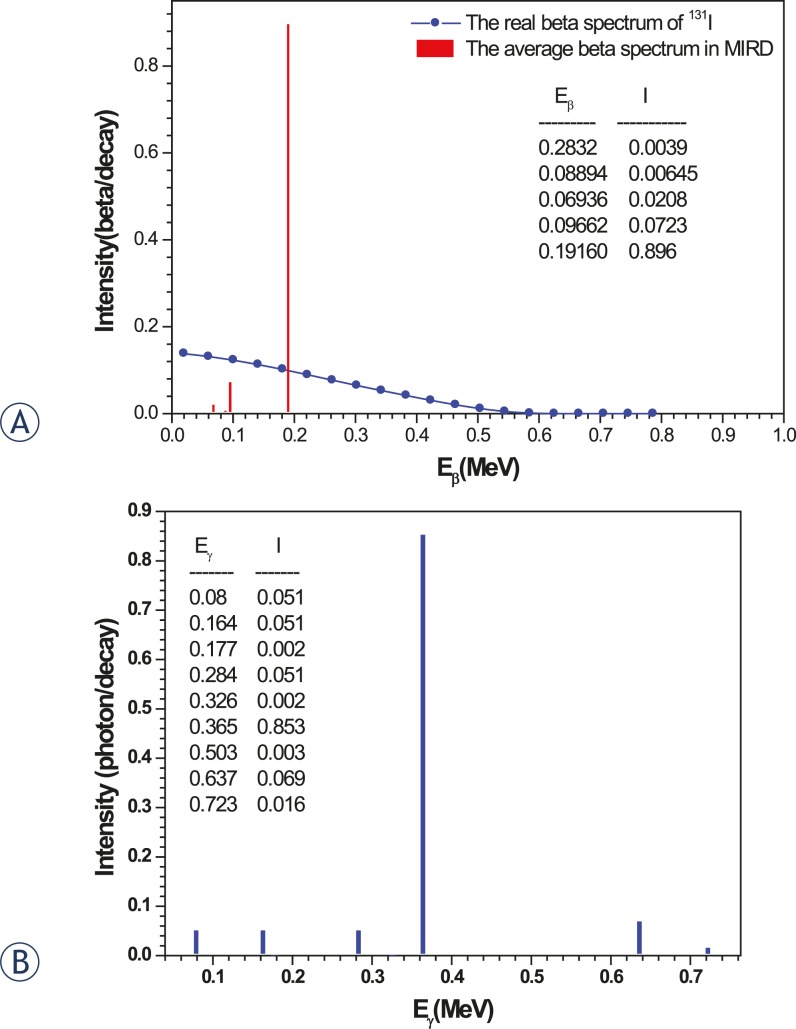
Radiation spectra of ^131^I radionuclide: a) the real beta spectrum and the average beta spectrum used in MIRD, b) the photons spectrum.

Figure 1A shows the real beta spectrum of ^131^I that we have used for our simulation, and the average beta spectrum used in MIRD, according to the Evaluated Nuclear Structure Data File (ENSDF) decay data. In the MIRD format, the beta spectrum includes 5 discrete lines, each representing the average beta energy and the yield for ^131^I beta radiations.^8^ As well as, the gamma spectrum is presented in Figure 1B.

The adult 70 kg human MIRD5 phantom has been used: the source organ was the thyroid gland with a uniform ^131^I distribution; the neck has been simulated with more detailed organs including skin, adipose layer under the skin, bone, spinal cord, thyroid lobes, and the remaining part as soft tissue. We have considered for soft tissue 1.05 g/ cm^3^ density and the ICRP composition.

As it is well known, the basic formula for absorbed dose rate used in MIRD formulation is:

[1] $$\frac{dD}{dt}=w\left(\sum_{i}n_{i}E_{i}\Phi_{i}\right)\frac{A}{m}$$

where *w* is a proportional constant, A is the radionuclide activity within the source organ, *n_i_* is the number of radiations with energy *E_i_* emitted per one decay, *Φ_i_* is the fraction of energy emitted in the source that is absorbed in the target organ, and *m* is the mass of the target. When the thyroid is considered both as source and target organ, the beta and gamma rays absorption fraction (*Φ_i_*) depends on thyroid volume.

We have selected *σ* as proper parameter to evaluate, by rewriting of Equation [1]:

[2] $$\frac{dD}{dt}=\sigma \frac{A}{m}\;\;\;\;\;\;;\;\;\;\;\;\;\;\;\sigma =w\left(\sum_{i}n_{i}E_{i}\Phi_{i}\right)$$

The activity administrated for hyperthyroidism and thyroid cancer therapy is varying inversely with *σ*. In many literatures, such as MIRDOSE code, *σ* is taken as a constant value 0.0313 (mGy g MBq^−1^ s^−1^), calculated by MC method for gamma rays and considering all beta energy deposited in a thyroid lobe of spherical shape, with fixed mass of 10 g. We have calculated the total (beta and gamma) energy deposition and *σ* for different volumes of thyroid lobes.

## Results and discussion

**FIGURE 2.**
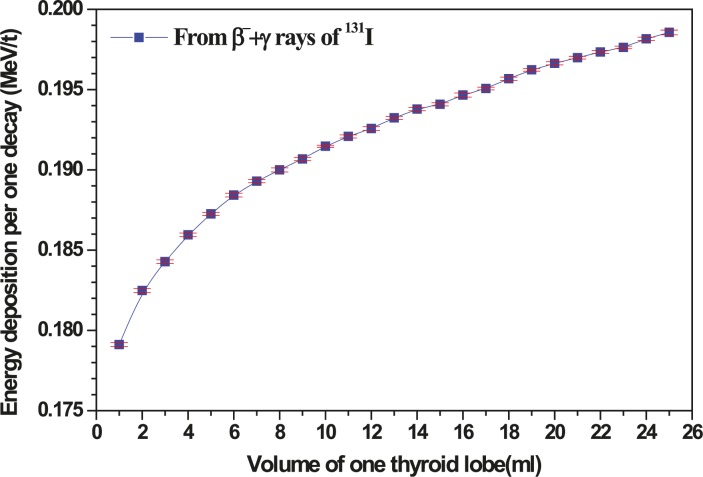
Variation of total energy deposition per decay against the volume of thyroid lobe.

Figure 2 shows the variation of the total energy deposition per decay of ^131^I for both beta and gamma rays against the volume of thyroid.

The total energy deposition per decay is the term in brackets $\left(\sum_{i}n_{i}E_{i}\Phi_{i}\right)$ and it increases by volume, because the increasing volume to surface ratio of ellipsoidal lobe causes the decrease of radiations fraction escaping from the lobe (Figure 2).

**FIGURE 3.**
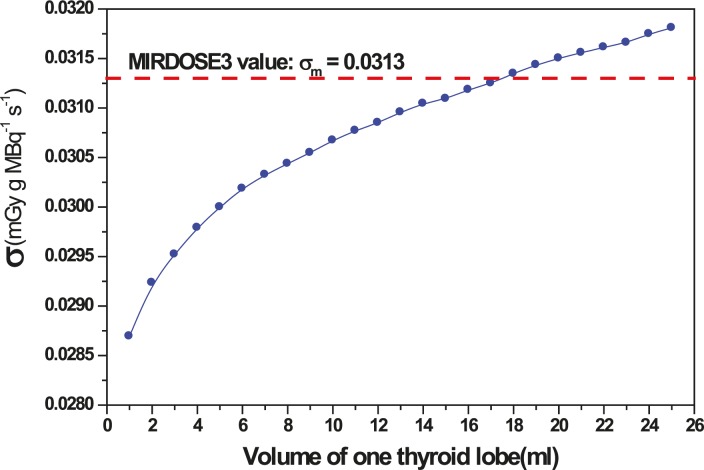
Influence of the thyroid lobe volume on value.

The calculated value of *σ* against the thyroid volume lobe has been presented in Figure 3. It can be seen that *σ* has a significant difference with the previous constant value, ranging from 10% to -1% when the lobe’s volume varies from 1 ml to 25 ml. For a 10 g lobe, our calculation shows about 2.2% difference with MIRDOSE3 *σ* value. This difference comes from two main sources: the first is the beta spectrum, as we have used the spectrum of ^131^I taken from a reference published by Eckerman *et al.* in 1994 in Health Physics^9^,^10^, with a mean beta energy of 0.1822 MeV per disintegration; the second is due to considering in our calculation the full beta and gamma transport in an ellipsoidal thyroid lobe (Figure 3).

We have used the photon energy deposition tally, called F6:p in MCNPX code, to calculate the photon energy deposition per unit of mass, in the other organs of the body, due to a decay in the source organ. It is clear that the result is proportional to the dose organ per one decay in the source.

**FIGURE 4.**
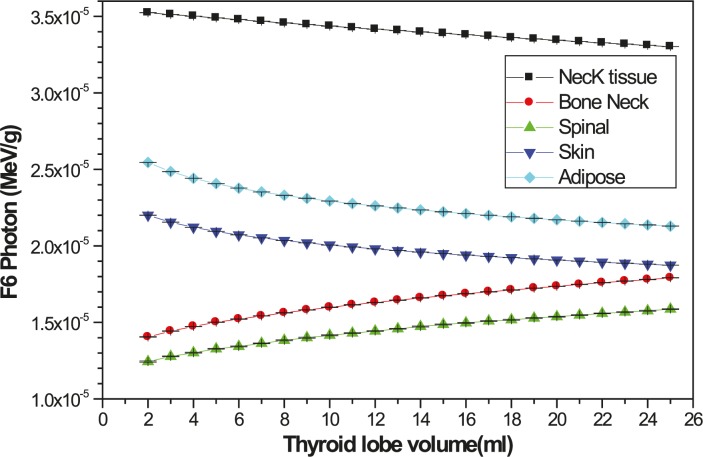
The variation of the energy deposition in other organs of neck respect to the thyroid lobe volume, per decay.

**FIGURE 5.**
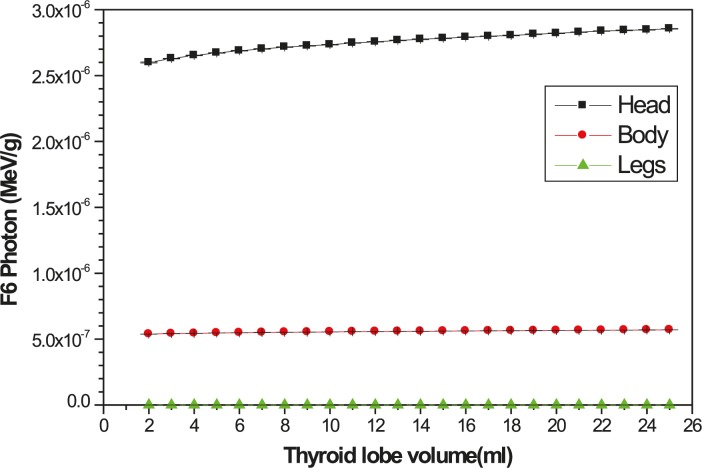
The variation of the energy deposition to head, body and legs respect to the thyroid lobe volume, per decay.

The energy deposition in other organs of neck as a function of the thyroid lobe volume per decay of ^131^I has been shown in Figure 4. As it is predictable, by increasing the lobe volume the dose in the bone and spinal cord increases but for other organs it decreases. The energy depositions per decay to organs far from the thyroid, including head, body and legs have been presented in Figure 5.

## Conclusions

The result shows that considering the lobe volume or mass has a significant effect over the absorbed dose calculation in thyroid gland. So, an accurate determination of the active volume of thyroid is very important in activity evaluation for radiomethabolic therapy by Iodine-131. As well as, according to our calculation, we suggest re-evaluating the *Φ_i_* value for gamma and beta sources when the source organ is the same as target and its volume or mass variation among different patients is considerable.

## References

[1] Traino AC, di Martino F, Lazzeri M, Stabin MG (2000). Influence of thyroid volume reduction on calculated dose in radioiodine therapy of Graves’ hyperthyroidism. Phys Med Biol.

[2] Becker DV (1989). Choice of therapy for Graves’ hyperthyroidism. N Eng J Med.

[3] Farrar JJ, Toft AD (1991). Iodine-131 treatment of hyperthyroidism. Clin Endocrinol Oxf.

[4] Vardar E, Erkan N, Bayol U, Yilmaz C, Dogan M (2011). Metastatic tumours to the thyroid gland: report of 3 cases and brief review of the literature. Radiol Oncol.

[5] Van Isselt JW, de Klerk JMH, Van Rijk PP, Van Gils APG, Polman LJ, Kamphuis C, Meijer R, Beekman FJ (2003). Comparison of methods for thyroid volume estimation in patients with Graves’ disease. Eur J Nucl Med Mol Imaging.

[6] Snyder W, Ford M, Warner G (1969). Estimates of absorbed fractions for monoenergetic photon sources uniformly distributed in various organs of a heterogeneous phantom: MIRD pamphlet no. 5. J Nucl Med.

[7] Waters LS (2002). MCNPX User’s Manual, version 2.3.0.

[8] http://www.orau.org/ptp/PTP%20Library/library/DOE/bnl/nuclidedata/MIRI131.htm

[9] Eckerman KF, Westfall RJ, Ryman JC, Cristy M (1994). Availability of nuclear decay data in electronic form, including beta spectra not previously published. Health Phys.

[10] Cember H, Johnson TE (2009). Introduction to health physics.

